# Correction: Activation of MAP3K DLK and LZK in Purkinje cells causes rapid and slow degeneration depending on signaling strength

**DOI:** 10.7554/eLife.86472

**Published:** 2023-02-13

**Authors:** Yunbo Li, Erin M Ritchie, Christopher L Steinke, Cai Qi, Lizhen Chen, Binhai Zheng, Yishi Jin

**Keywords:** Mouse

Li Y, Ritchie EM, Steinke CL, Qi C, Chen L, Zheng B, Jin Y. 2021. Activation of MAP3K DLK and LZK in Purkinje cells causes rapid and slow degeneration depending on signaling strength. *eLife* **10**:e63509. doi: 10.7554/eLife.63509.

Published 21 January 2021

We are correcting two images in the published Figure 4A and Figure 5G. During the making of enlarged images of Purkinje cells, we inadvertently used a cerebellum image of Map3K12^fl/fl^; Hipp11-LZK(iOE)/+ (P60) to produce an enlarged image of Purkinje cells in Hipp11-LZK(iOE)/+shown in Figure 4A (upper row, P60). In Figure 5G, we inadvertently used a cerebellum image of Hipp11-LZK(iOE)/+ (P120) to produce an enlarged image of Purkinje cells in Pvalb^Cre/+^(P120) (left image). We have now made the correction using the cerebellum images of correct genotypes. The correction of images does not in any way change our conclusions. There is no other change in the main text or figure legends of the paper. We thank the readers for bringing to our attention the problematic images and apologize for not catching the errors prior to publication.

The corrected Figure 4 is shown here:

**Figure 1:**
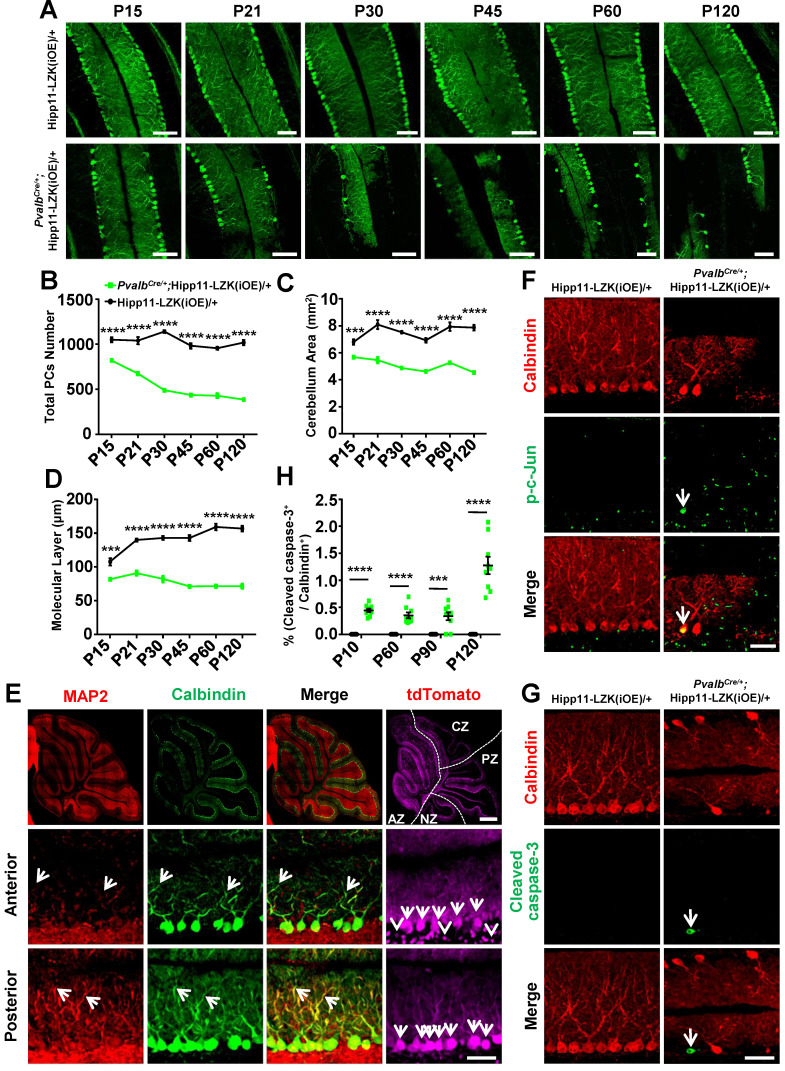


The originally published Figure 4 is shown for reference:

**Figure 2:**
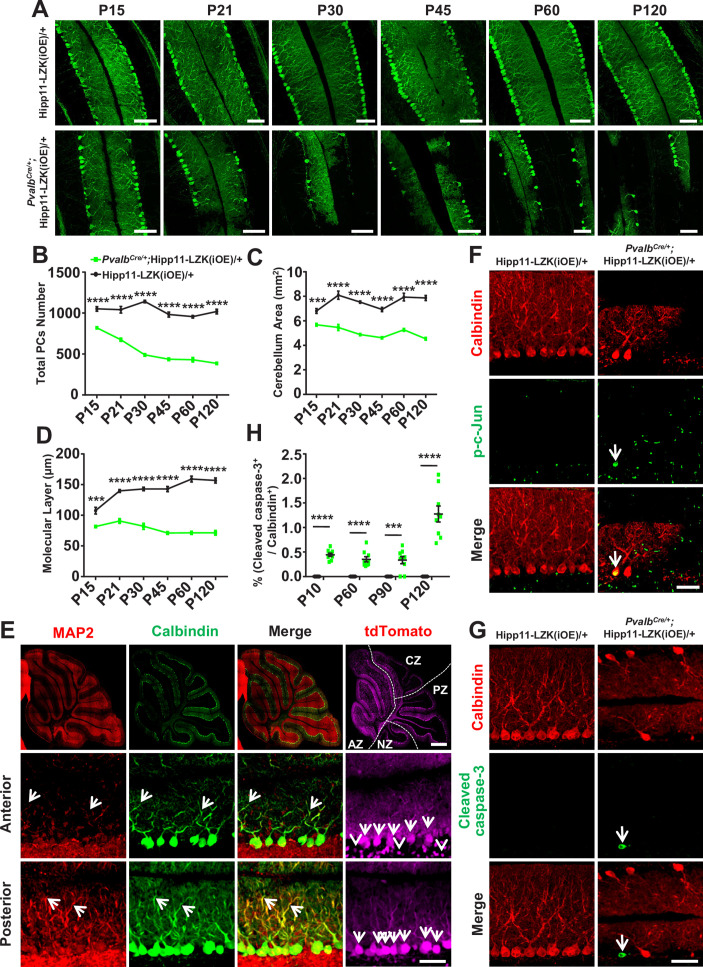


The corrected Figure 5 is shown here:

**Figure 3:**
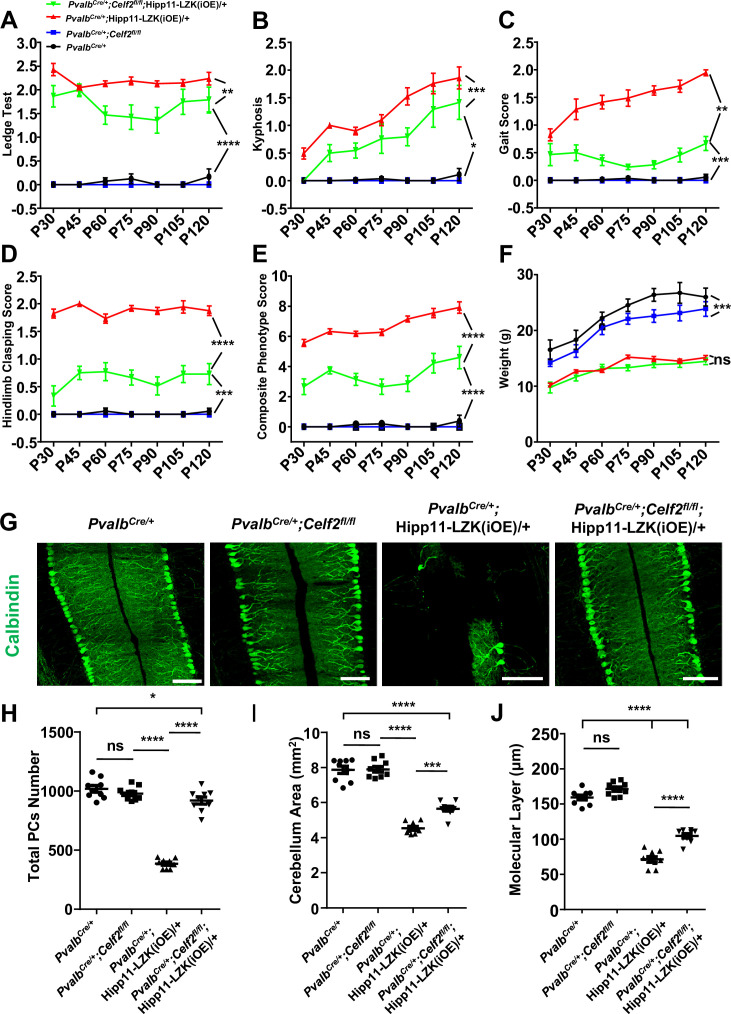


The originally published Figure 5 is shown for reference:

**Figure 4:**
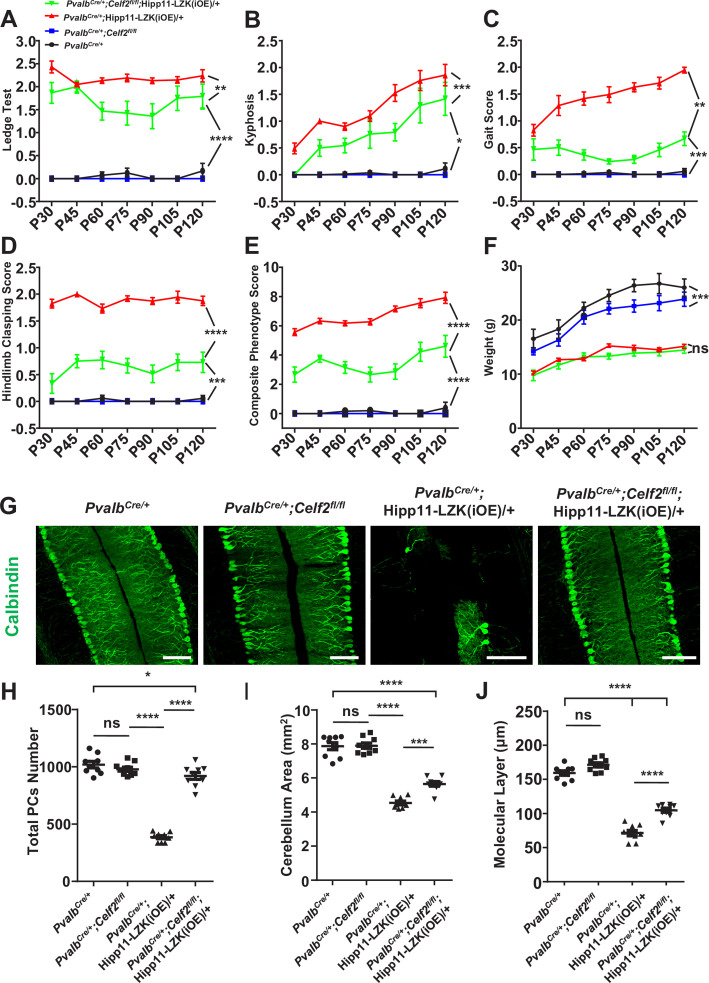


In addition, we found a production error that resulted in missing of the red boxes on the H&E images (left panels in Figure 1A) and faint white boxes on the panels of Pvalb^Cre/+^; Hipp11-LZK(iOE)/+in Figure 6C. These are now corrected by the publisher.

The corrected Figure 1 is shown here:

**Figure 5:**
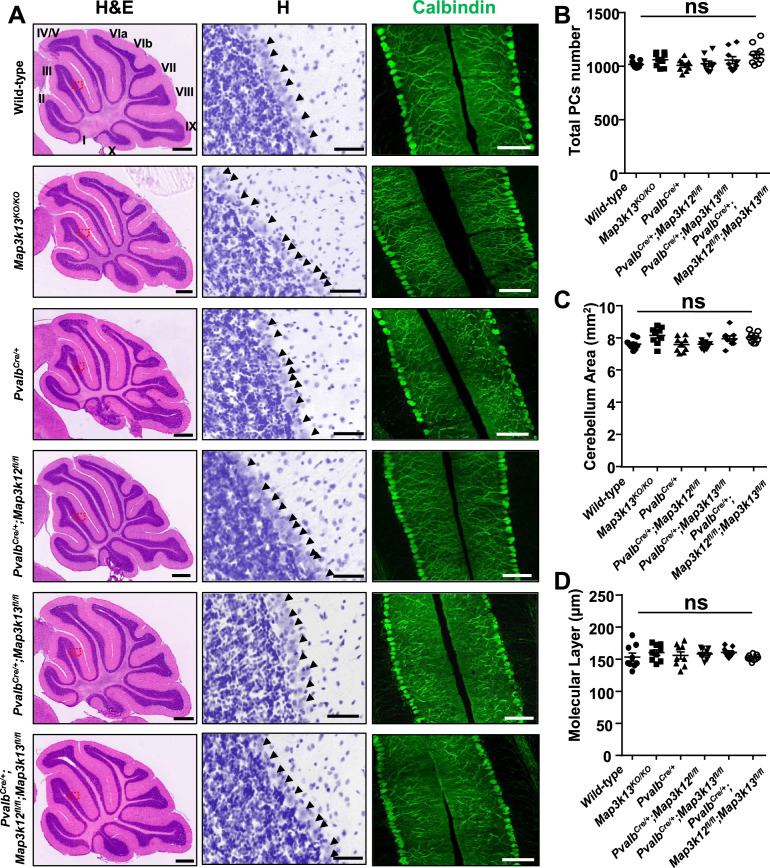


The originally published Figure 1 is shown for reference:

**Figure 6:**
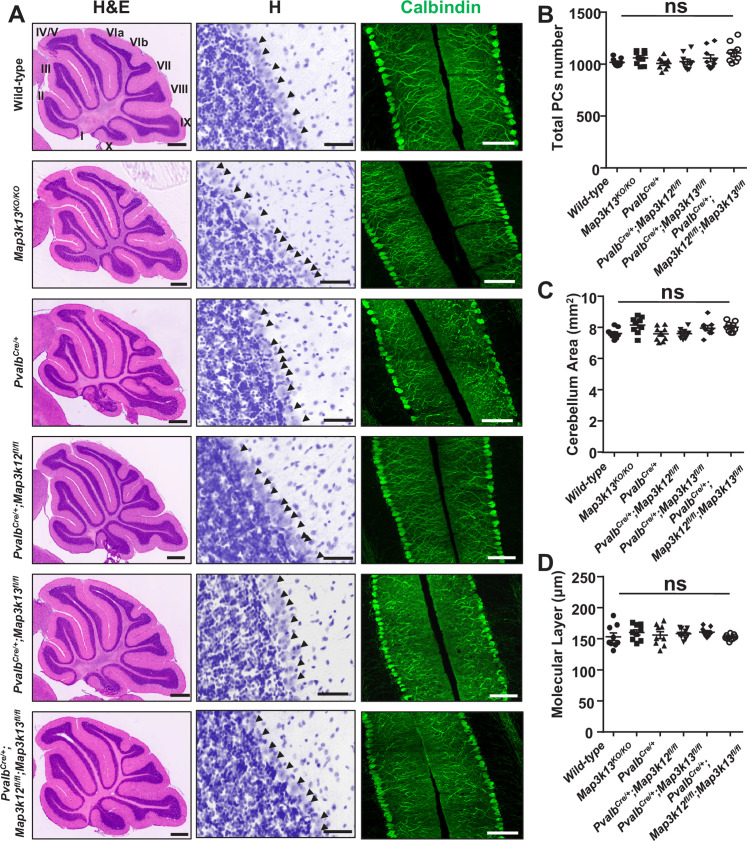


The corrected Figure 6 is shown here:

**Figure 7:**
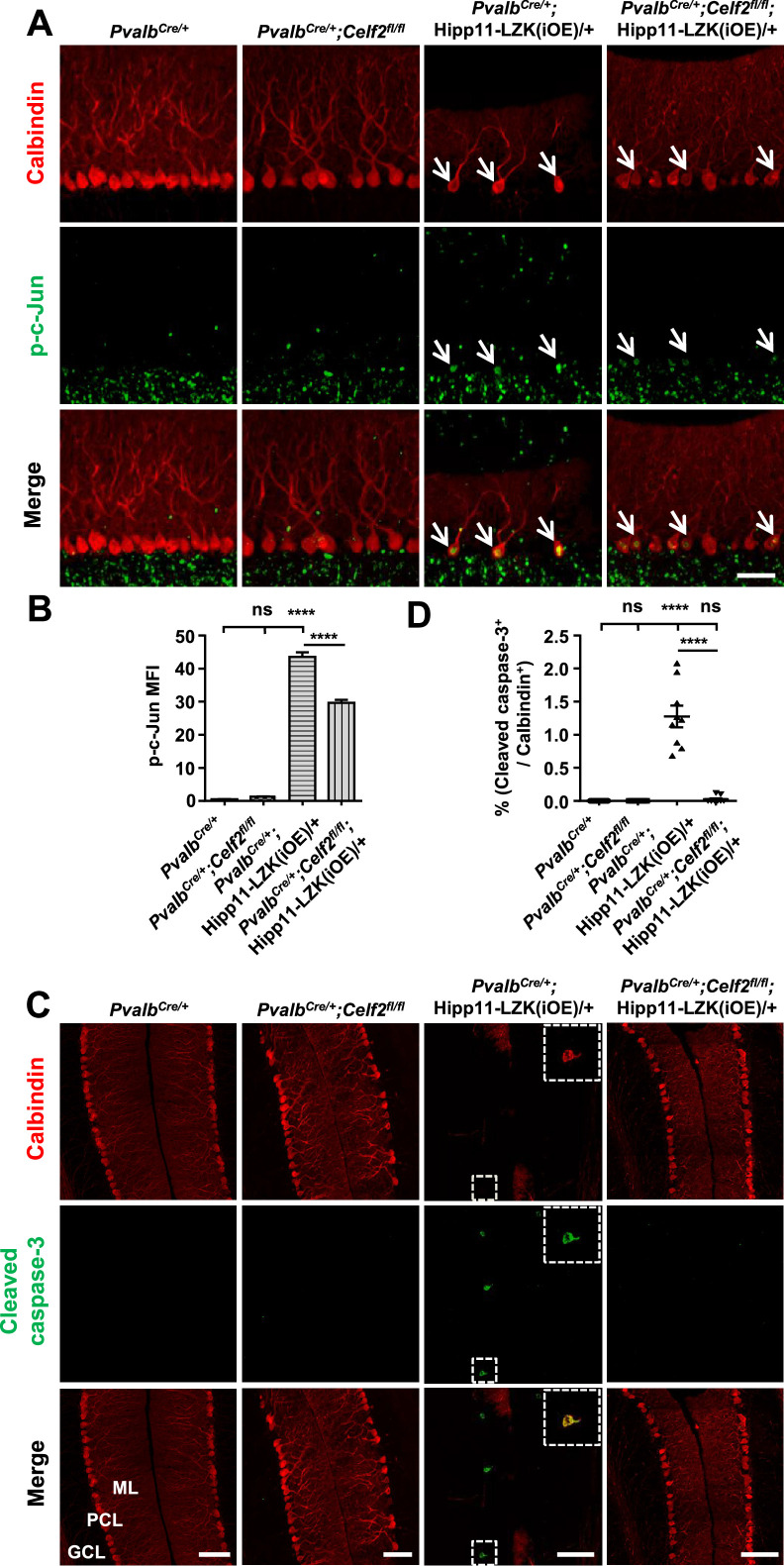


The originally published Figure 6 is shown for reference:

**Figure 8:**
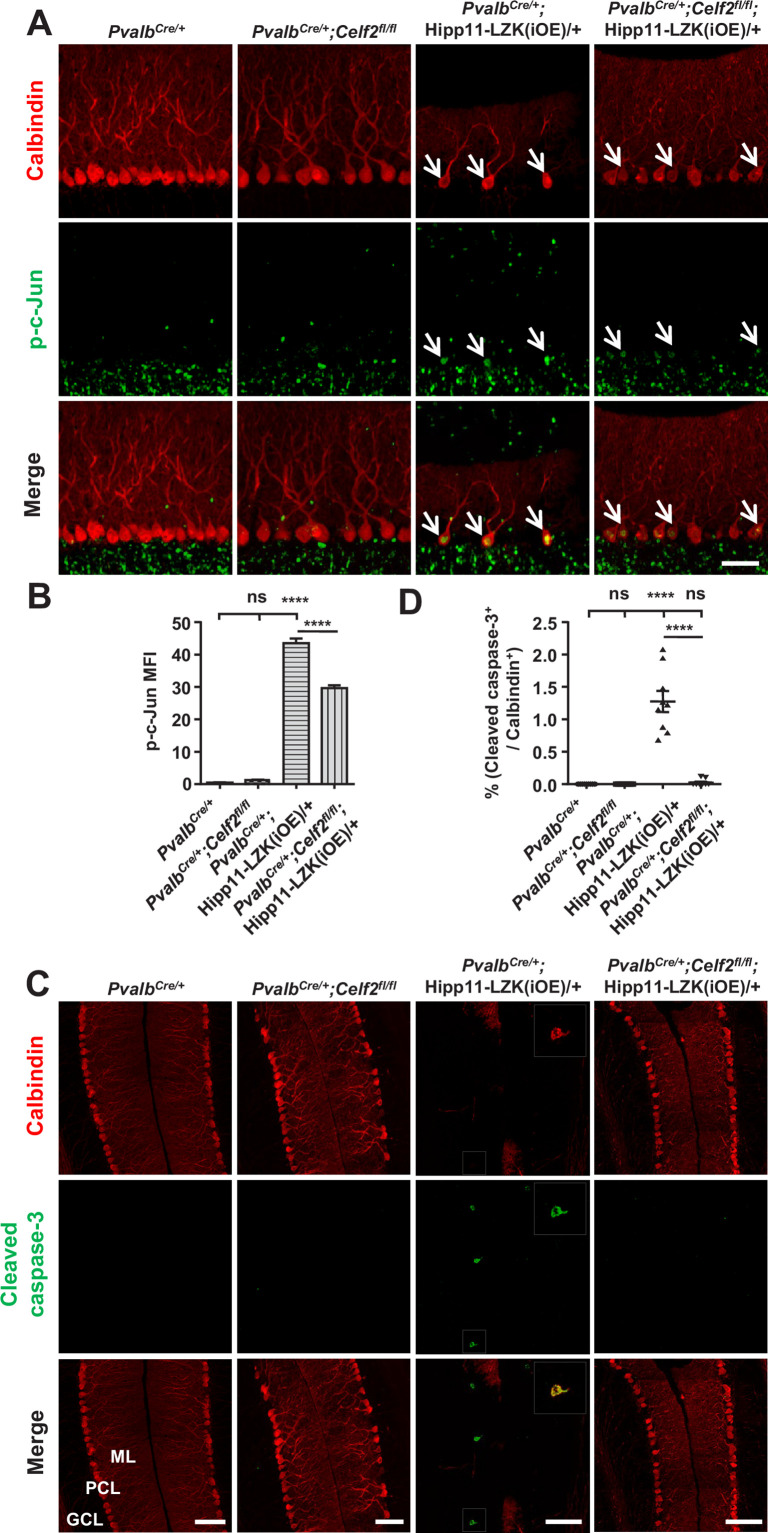


The article has been corrected accordingly

